# Humic Acid Composition and Characteristics of Soil Organic Matter in Relation to the Elevation Gradient of Moso Bamboo Plantations

**DOI:** 10.1371/journal.pone.0162193

**Published:** 2016-09-01

**Authors:** Hsueh-Ching Wang, Chiao-Ying Chou, Chyi-Rong Chiou, Guanglong Tian, Chih-Yu Chiu

**Affiliations:** 1 Biodiversity Research Center, Academia Sinica, Nankang, Taipei 11529, Taiwan; 2 Biodiversity Research Center, National Taiwan University, Taipei 10617, Taiwan; 3 School of Forestry and Resource Conservation, National Taiwan University, Taipei 10617, Taiwan; 4 Environmental Monitoring and Research Division, Monitoring and Research Department, Metropolitan Water Reclamation District of Greater Chicago (MWRD), Lue-Hing R&D Laboratory, 6001 W. Pershing Road, Cicero, IL 60804, United States of America; Tennessee State University, UNITED STATES

## Abstract

Studying the influence of climatic and/or site-specific factors on soil organic matter (SOM) along an elevation gradient is important for understanding the response of SOM to global warming. We evaluated the composition of SOM and structure of humic acids along an altitudinal gradient from 600 to 1400 m in moso bamboo (*Phyllostachys edulis*) plantations in central Taiwan using NMR spectroscopy and photometric analysis. Total organic C and total nitrogen (N) content increased with increasing elevation. Aromaticity decreased and ΔlogK (the logarithm of the absorbance ratio of humic acids at 400 and 600 nm) increased with increasing elevation, which suggests that SOM humification decreased with increasing elevation. High temperature at low elevations seemed to enhance the decomposition (less accumulation of total organic C and N) and humification (high aromaticity and low ΔlogK). The alkyl-C/O-alkyl-C (A/O-A) ratio of humic acids increased with increasing elevation, which suggests that SOM humification increased with increasing elevation; this finding was contrary to the trend observed for ΔlogK and aromaticity. Such a discrepancy might be due to the relatively greater remaining of SOM derived from high alkyl-C broadleaf litter of previous forest at high elevations. The ratio of recalcitrant C to total organic C was low at low elevations, possibly because of enhanced decomposition of recalcitrant SOM from the previous broadleaf forest during long-term intensive cultivation and high temperature. Overall, the change in SOM pools and in the rate of humification with elevation was primarily affected by changes in climatic conditions along the elevation gradient in these bamboo plantations. However, when the composition of SOM, as assessed by NMR spectroscopy and photometric analysis was considered, site-specific factors such as residual SOM from previous forest and intensive cultivation history could also have an important effect on the humic acid composition and humification of SOM.

## Introduction

Soil organic matter (SOM) is the largest terrestrial pool of carbon (C), with a greater C content (1500–2400 Pg C) than the sum of atmospheric C (829 Pg C) and terrestrial vegetation C (450–650 Pg C) [1]. The carbon dioxide released to the atmosphere through decomposition of dead organic matter in litter and soil plays an important role in the global C cycle [2, 3]. The substantial pool of C in soil is sensitive to changes in climate and the local environment, and increasing warming might affect soil C flux by altering vegetation productivity, SOM input and soil microbial activity [4, 5]. Understanding the interactions between SOM and climatic conditions is critical for modeling global and local C balance.

Temperature decreases with increasing elevation in a montane ecosystem, a region, which is considered to be vulnerable to global warming [6–8]. A montane system provides climatic-dominated observations over short spatial distances along an elevation gradient. Soil microbial communities and activity are susceptible to changes in temperature, soil moisture, substrate quantity and quality. The interactions of climatic factors along a climosequence play an important role in determining soil characteristics and the C pool in montane systems [9–11]. Decreases in temperature with increasing elevation can reduce soil microbial activity and organic matter decomposition [5, 9]. High-elevation regions could be more sensitive to increased temperature, and an increase in temperature could greatly enhance the rate of decomposition [12].

However, the pattern of SOM along an elevation gradient and the factors involved are still not well understood. In general, the turnover rate of SOM has been positively correlated with mean annual temperature in an elevation gradient, resulting in the accumulation of SOM at high elevations [8, 13]. Djukic et al. [6] found that vegetation composition, C inputs and litter quality rather than climatic factors altered soil C groups along an elevation gradient. Site-specific factors such as stand productivity, vegetation management and land use history could also play an important role in the accumulation of soil C [14]. This spatial variation of SOM, which could be controlled by climatic or site-specific factors along an elevation gradient, can affect soil respiration and C budget [10].

In addition, composition and structure of SOM along the elevation gradient reflect soil microbial activity and SOM decomposition rate [15]. During humification, organic materials are transformed to humic substances, resulting in SOM with a significantly altered structure compared with the original plant materials [16]. Environmental characteristics, such as temperature and precipitation, change along an elevation gradient, and this could affect the process of humification and the formation of humic substances in the soil. Solid-state ^13^C nuclear magnetic resonance spectroscopy with cross-polarization and magic-angle spinning (CP-MAS ^13^C NMR) has been used to determine the functional groups and humification of SOM [17]. For example, Rossi [18] used NMR spectroscopy to characterize the change in composition and structure of SOM after fire disturbance. Faz Cano et al. [19] indicated that O-alkyl-C content increased but aromatic-C content decreased with increasing elevation. Using NMR spectroscopy to determine the composition and structure of SOM could be helpful in understanding the effects of climosequence and/or site-specific environment on C sequestration along an elevation gradient.

Bamboo occupies approximately 1% of the global forest area in tropical and subtropical regions, and the total area forested in bamboo increased by 1.6 million ha between 1990 and 2010 [20]. Moso bamboo (*Phyllostachys edulis*) is the most important bamboo species in parts of Asia. Moso bamboo covers 3 million hectares in China and was introduced to central Taiwan more than 100 years ago [21, 22]. Bamboo spreads laterally due to a unique root rhizome system from which shoots and culms emerge. Because of this rhizome system, bamboo grows quickly and is one of the fastest-growing plants in the world [23]. The intensive management of bamboo plantations, including regular removal of bamboo shoots and culms, tillage, and fertilization, can reduce total and labile SOM stocks and increase soil CO_2_ efflux [22, 24, 25]. The easily decomposed herbaceous bamboo litter maintains soil microbial functional diversity and community structure [26, 27].

Huang et al. [22] reported that soil soluble organic C and total N content increased with increasing elevation in bamboo plantations, but SOM humification in the bamboo plantations and composition of humic acids along an elevation gradient were not studied. The objective of this study was to evaluate the effect of climosequence on the humic acid composition and humification of SOM in bamboo plantations using photometric analysis and NMR spectroscopy, together with acid-hydrolysis.

## Materials and Methods

### Study site

**Fig 1:**
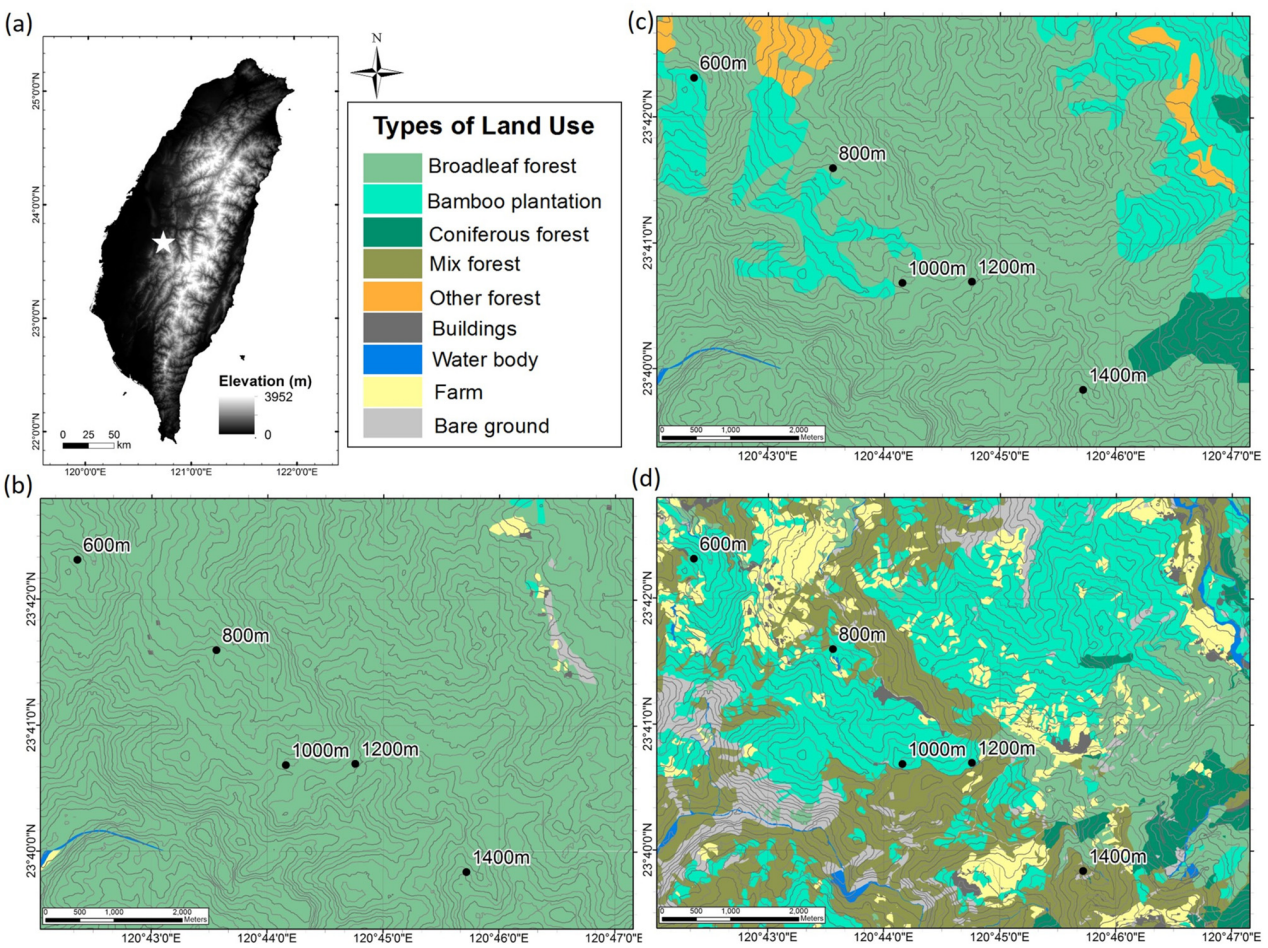
Maps of land use changes in five bamboo plantations. (a) The location of the study site overlaid on an elevation gradient map. (b) Land use in 1904. (c) Land use in 1956. (d) Land use in 2006. The mixed forest indicates that bamboo plantation mixed with broadleaf forest, and coniferous forest. The coordinates recorded were (120°42’22” E, 23°42’19” N) at 600 m, (120°43’32” E, 23°41’36” N) at 800 m, (120°44’10” E, 23°40’41” N) at 1000 m, (120°44’44” E, 23°40’42” N) at1200 m and (120°45’44” E, 23°39’50” N) at 1400 m asl.

This study was carried out at Mt. Da-an (120°42’ E, 23°42’ N), Zhushan, Nantou County, Taiwan. The study was permitted by Mr. G. T. Wu, manager of the Dinglin Forestry Production Cooperative. The field studies did not involve endangered or protected species. This study site has a humid subtropical climate with the mean annual precipitation ranging from 2210 to 2600 mm. The soils with a 30−50 cm depth, were acidic, sandy loam to loam, and classified as Entisols based on soil taxonomy [22]. The natural vegetation in the region was broadleaved forest, which was replaced with bamboo plantations following settlement and development [28]. Based on the record from weather stations at 84 m, 800 m, 1150 m, 1755 m and 2413 m asl, we established a negative correlation between annual mean air temperature and elevation. Based on the temperature-elevation correlation, the annual mean air temperature was estimated as 20.3°C at 600 m and 16.1°C at 1400 m with a decrease of 0.52°C per 100 m elevation gain. Five moso bamboo plantations at 600, 800, 1000, 1200, and 1400 m were selected for soil sampling (Fig 1).

Moso bamboo, a temperate species of giant timber bamboo, can reach heights of up to 28 m in 40 to 50 days [21]. In the study region, moso bamboo was distributed at altitudes from 500 to 1500 m [22]. The bamboo density of the study plantations was 5333−6900 culms ha^-1^, the mean height of bamboo ranged from 8.8−12.6 m, and the mean bamboo age ranged from 2.5−4.2 years [29]. Altitudinal effects on bamboo growth parameters, such as diameter, height, height at crown base, and biomass, were known to be positive from 600 m to 1400 asl in this area [29]. The soils at low-elevation sites were more disturbed by activities such as harvesting of bamboo shoots in winter, removal of understory plants, and fertilizing than the high-elevation sites.

To obtain the land use history of the study site, we used Taiwan Fort Maps, printed in 1904 (S1 Fig), First Forest Resource and Land Use Inventory Maps, printed in 1956 (S2 Fig), and the Second Time Land Use Investigation Plan in Taiwan, printed in 2006 [30, 31, 32] (S3 Fig). The Maps shows land cover types, land use conditions, and forest stand-size delineations. Remotely sensed images and aerial photos were primarily used to establish the primary linear map and preliminary land use estimates [33]. Based on these records, we could be sure that in 1904, the study site was broadleaf forest. By 1956, low-elevation sites (600 m, 800 m and 1000 m) had been replaced by bamboo plantations. However, high-elevation sites (1200 m and 1400 m) remained as broadleaf forest until 1956. Bamboo plantations at high elevations were established later than those at low elevations, and they expanded largely between 1956 and 2006. By 2006, bamboo plantations occurred all over the study site (Fig 1).

### Soil sampling and analysis

Five plots (replications) were randomly marked out in each of the five bamboo plantations in an elevation gradient. Each plot had a size of 50 × 50 m, and the distance between two plots was at least 50 m. Soil samples were taken at the surface (0–10 cm) in January 2012 using a soil corer (8 cm in diameter and 10 cm deep). Five soil cores were taken randomly in each plot and combined as a composite sample for that plot. Visible roots, litter residue, and soil fauna were removed before sieving. Soil samples were air-dried, ground through a 2-mm sieve, and kept at 4°C prior to analysis.

The pH was determined at a 1:1 soil/water ratio. Total organic C and total N were measured by the combustion method with the use of a Fisons NA1500 elemental analyzer (ThermoQuest Italia, Milan, Italy). Soil subsamples were oven-dried at 105°C to determine soil water content (w/w, %).

### Extraction of soil humic acids

The humic portion of the soil was extracted with 0.1 M NaOH after 4 h of shaking in a soil to extractant ratio of 1:10 under N_2_ atmosphere. The alkaline supernatant was centrifuged at 15,000 x *g* for 20 min and acidified to pH 1.0 with 6 N HCl at room temperature for 12–16 h. The supernatant (fulvic acids) was centrifuged from coagulated materials (humic acids) at 5,500 x *g* for 15 min. The humic acids were purified with a 0.1 M KOH and 0.3 M KCl mixture and centrifuged at 10,000 x *g* for 10 min. The supernatant was acidified to pH 1.0 with 6 N HCl and centrifuged at 5,500 x *g* for 15 min. The coagulated materials were shaken with 0.1 M HCl: 0.3 M HF overnight and centrifuged at 5,500 x *g* for 15 min. The humic acid fractions were condensed and freeze-dried for ^13^C CP-MAS NMR spectroscopy.

### Photometric analysis

We used photometric analysis to obtain the absorbance property of the humic acids (ΔlogK), which is defined as the logarithm of the absorbance ratio of humic acids at 400 and 600 nm, i.e. ΔlogK = log(A_400_/A_600_) [34]. The ΔlogK is considered to be an inverse index of the condensation of the aromatic network in humic acid macromolecules and the degree of humification. One gram of air-dried soil was extracted with 30 ml 0.1 N NaOH and shaken for 30 min at 100°C. Samples were extracted with 2 ml Na_2_SO_4_ and centrifuged at 10,000 x *g* for 15 min. The precipitates were dissolved in 20 ml 0.1 N NaOH, and the humic acid fraction was obtained by acidification of 100 ml of extractant with 1 ml concentrated H_2_SO_4_ (98%) as precipitates. After centrifugation, the humic acid precipitates were dissolved in 30 mL 0.01 N NaOH to measure the absorbance of the humic acids at 400 and 600 nm by use of a spectrophotometer (Hitachi U-2000) [16].

### NMR spectroscopy

The C functional groups were examined in the freeze-dried humic acid samples by use of solid-state ^13^C CP-MAS NMR spectroscopy (BRUKER DSX 400-MHz solid-state NMR, Germany) with a 7-mm-diameter sample tube. Data acquisition conditions were as follows: spectrometer frequency 100.46 MHz, spectra width 20,000 Hz, spinning speed 7,000 Hz, contact time 6 ms, and pulse delay time 1 sec. Scans of 8,200 were collected [35, 36]. The contributions of C functional groups were divided into four chemical-shift areas: 0–50 ppm (alkyl-C), 50–110 ppm (O-alkyl-C), 110–165 ppm (aromatic-C), and 165–190 ppm (carboxyl-C) [17]. The relative intensities of chemical-shift areas were determined by the integration of ^13^C CP-MAS NMR spectra over given chemical-shift ranges to the total spectrum area. The aromaticity was calculated as the ratio of aromatic-C (110–165 ppm) to the sum of alkyl-C, O-alkyl-C and aromatic-C (0–165 ppm) [37]. The ratio of alkyl-C to O-alkyl-C (A/O-A) and aromaticity were used to evaluate the degree of humification [38].

### Determination of acid-hydrolysable and recalcitrant C

Acid hydrolysis is a chemical fractionation method used to separate acid-hydrolysable (labile) and recalcitrant SOM (unhydrolysable) [39]. This study used two-step acid hydrolysis with sulfuric acid (H_2_SO_4_) as the extractant to quantify acid-hydrolysable and recalcitrant SOM [40]. A 0.5-g soil sample was hydrolyzed with 20 ml of 5 N H_2_SO_4_ at 105°C for 30 min in Pyrex tubes with Allihn condensers. Centrifugation and decantation were used to collect the hydrolysate. Then, 20 ml of de-ionized water was used to flush the residue, and the extract was added to the previous hydrolysate. The C in the hydrolysate was termed acid-hydrolysable pool-I C (AHPI-C). The remaining residue was hydrolyzed with 2 ml of 26 N H_2_SO_4_ overnight at room temperature under continuous shaking. The concentration of samples was reduced to 2 N by dilution and was hydrolyzed for 3 h at 105°C. The residue was flushed with 26 ml de-ionized water and added to the hydrolysate. The second hydrolysate was termed acid-hydrolysable pool-II C (AHPII-C). The remaining residue was washed with 30 ml de-ionized water and dried at 60°C in a pre-weighed crucible and termed the recalcitrant pool (RP-C). The AHPI-C and AHPII-C were measured with a total organic C analyzer (Model 1010, O.I. Analytical, Texas), while the RP-C was determined by a Fisons NA1500 elemental analyzer.

### Statistical analysis

One-way ANOVA was used to test the significance of altitudinal effects on soil properties, ^13^C NMR functional groups, and acid-hydrolysable and recalcitrant C pools. Tukey’s HSD comparisons were performed to determine the significance of difference between elevations. A p-value < 0.05 was considered statistically significant. All statistical analyses were performed with the Minitab 16 Statistical Software (Minitab Inc., PA, USA).

## Results

### Soil basic characteristics

The soil total organic C and N in the bamboo plantation increased with increasing elevation (*p* ≤ 0.001) and peaked at 1200 and 1400 m (Table 1). The soil C/N ratios remained unchanged along the plantation elevation gradient (*p =* 0.066). In general, the soil pH was strongly acidic (3.72–4.16) in the bamboo plantations.

**Table 1:** Soil water content, pH, total carbon (TC) and total nitrogen (TN), and C:N ratio (C/N) in surface soil (0–10 cm) in 5 moso bamboo plantations along an elevation gradient.

Elevation (m)	Soil water content (%)	pH (H_2_O)	TC (%)	TN (%)	C/N
600	74.2 ± 2.9^a^	3.72 ± 0.03	2.55 ± 0.25 ^c^	0.25 ± 0.02 ^d^	10.2 ± 0.48
800	69.4 ± 1.9^ab^	3.77 ± 0.09	3.32 ± 0.15 ^c^	0.32 ± 0.03 ^c^	10.4 ± 0.46
1000	65.0 ± 3.5^bc^	3.77 ± 0.08	4.31 ± 0.15 ^b^	0.42 ± 0.02 ^b^	10.4 ± 0.16
1200	59.0 ± 3.6^d^	3.76 ± 0.11	5.95 ± 0.39 ^a^	0.53 ± 0.04 ^a^	11.2 ± 0.39
1400	62.5 ± 6.0^cd^	4.16 ± 0.19	6.07 ± 0.79 ^a^	0.52 ± 0.04 ^a^	11.6 ± 1.45
*p*-value	< 0.001	> 0.05	< 0.001	< 0.001	0.066

Values within a column followed by different letters are significantly different at *p* = 0.05.

### Patterns of ^13^C NMR functional groups and photometric analysis

**Fig 2:**
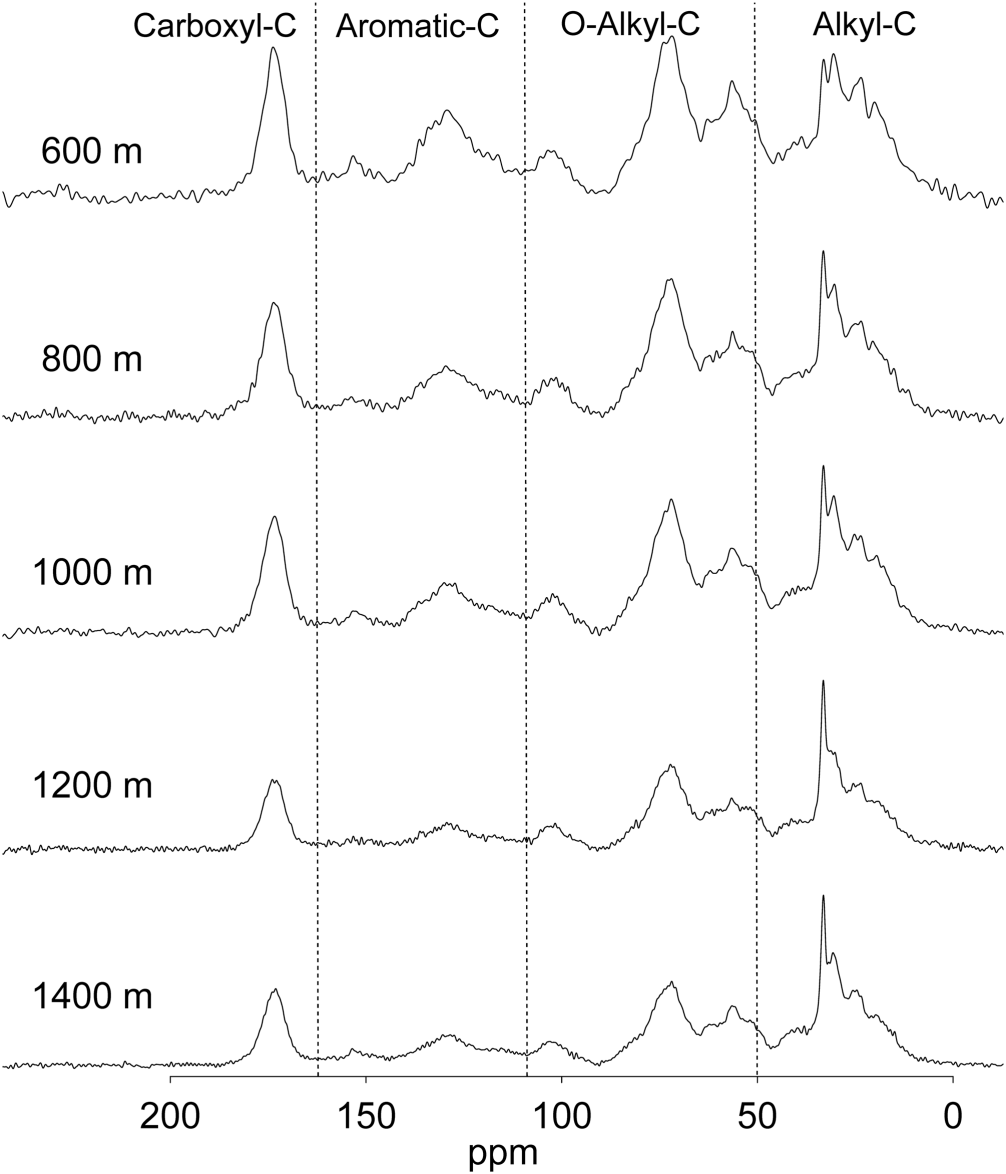
The ^13^C NMR spectra of soil humic acids in five bamboo plantations at 600, 800, 1000, 1200 and 1400 m asl. The chemical shift regions were 0–50 ppm (alkyl-C), 50–110 ppm (O-alkyl-C), 110–165 ppm (aromatic-C), and 165–190 ppm (carboxyl-C).

The ^13^C NMR spectra of soil humid acids were divided into alkyl-C, O-alkyl-C, aromatic-C, and carboxyl-C functional groups (Fig 2). In the bamboo plantations, O-alkyl-C was the major component (37.4–39.0%), followed by alkyl-C (26.8–35.1%), aromatic-C (14.7–23.4%), and carboxyl-C (11.4–12.6%). The proportion of aromatic-C and alkyl-C differed significantly along the elevation gradient (*p* < 0.05), while that of carboxyl-C and O-alkyl-C was similar (*p* ≥ 0.21) (Table 2). The proportion of aromatic-C decreased with increased elevation and was lowest (14.66%) at 1200 m, whereas alkyl-C increased with increasing elevation and peaked (35.09%) at 1200 m. The aromaticity index decreased along the elevation gradient and was lowest (16.54) at 1200 m, whereas the A/O-A ratio increased with increasing elevation and peaked at 1200 (0.91) and 1400 m (0.92). The ΔlogK of the soil humic acids was significantly different (*p =* 0.031) among the plantations and was higher at high elevations (1200 and 1400 m) compared with low elevations (600, 800 and 1000 m) (Table 2).

**Table 2:** Proportions of ^13^C NMR functional groups, photometric analysis of humic acids and degree of soil organic matter (SOM) humification in five bamboo plantations along an elevation gradient.

Elevation	Functional groups (%)				A/O-A ratio	Aromaticity	ΔlogK
	Alkyl-C	O-alkyl-C	Aromatic-C	Carboxyl-C			
600	26.84 ^c^	37.35	23.44 ^a^	12.37	0.72 ^b^	26.76 ^a^	0.589 ^ab^
800	29.60 ^c^	39.04	18.78 ^b^	12.59	0.76 ^ab^	21.48 ^b^	0.574 ^b^
1000	30.82 ^bc^	38.63	18.26 ^bc^	12.30	0.80 ^ab^	20.82 ^b^	0.589 ^ab^
1200	35.10 ^a^	38.88	14.66 ^c^	11.37	0.91 ^a^	16.54 ^c^	0.644 ^a^
1400	34.74 ^ab^	37.79	15.42 ^bc^	12.07	0.92 ^a^	17.53 ^bc^	0.622 ^ab^
*p*-value	0.002	0.493	0.001	0.206	0.018	0.001	0.031

Values within a column followed by different letters are significantly different at *p* = 0.05.

### Acid-hydrolysable and recalcitrant pools of SOM

The concentrations of soil acid-hydrolysable C (AHPI-C and AHPII-C) and recalcitrant C (RP-C) all increased with increasing elevation of bamboo plantations (*p* < 0.001) and peaked at 1200 m (Table 3). The ratio of AHPII-C to total organic C (AHPII-C/TC) decreased with increasing elevation, and was the lowest at 1400 m. The ratio of RP-C to TC (RP-C/TC) increased along the elevation gradient and peaked at 1200 m.

**Table 3:** Carbon content in acid-hydrolysable pool-I (AHPI-C) and II (AHPII-C) and recalcitrant pool carbon (RP-C) in five bamboo plantations along an elevation gradient.

Elevation	AHPI-C (g C kg^-1^)	AHPII-C (g C kg^-1^)	RP-C (g C kg^-1^)	AHPI-C/TC	AHPII-C/TC	RP-C/TC
600	12.38 ^c^	3.77 ^c^	12.74 ^c^	0.486	0.148 ^a^	0.500 ^c^
800	16.36 ^c^	4.18 ^c^	19.24 ^bc^	0.492	0.126 ^bc^	0.579 ^bc^
1000	21.09 ^b^	5.85 ^b^	24.43 ^b^	0.490	0.136 ^ab^	0.565 ^c^
1200	28.13 ^a^	7.10 ^b^	48.07 ^a^	0.473	0.120 ^c^	0.813 ^a^
1400	27.63 ^a^	6.08 ^a^	44.80 ^a^	0.457	0.100 ^d^	0.731 ^ab^
*p*-value	< 0.001	< 0.001	< 0.001	0.453	< 0.001	< 0.001

Values within a column followed by different letters are significantly different at *p* = 0.05.

## Discussion

The temperature varied along the elevation gradient, this may have affected soil total organic C and total N content in the moso bamboo plantations of different elevations. The higher soil total organic C and N content at high elevations (1200 and 1400 m) compared with low elevations (600, 800 and 1000 m) was primarily due to the decrease in SOM decomposition with increasing elevation because low temperature at high elevations can restrict microbial activity [6, 8].

The major component of humic acids determined by NMR spectroscopy in the bamboo plantations was O-alkyl-C, which was dominated by hydrolysable polysaccharides (cellulose and hemicellulose). Alkyl-C, which contains high concentrations of recalcitrant substances such as surface waxes, lipids, cutins, and plant litter resins, was the second greatest component of humic acids. Similar results were found in moso bamboo plantations in China [24]. The aromatic-C content, which was primarily composed of lignin and tannins, was considered highly recalcitrant and increased with humification [41, 42]. In general, aromaticity can be used as an index of SOM humification, and higher values indicate the high humification [37, 42]. The ΔlogK is also considered an index of SOM humification, and higher values indicate the lower humification [34]. Thus, the negative trend in aromaticity and the positive trend in ΔlogK with elevation increase indicated that the degree of SOM humification was greater in low-elevation soils compared with high-elevation soils. Therefore, high microbial activity supported by high temperatures at lower elevations could increase the rate of decomposition and intensify the degree of humification.

Significantly low concentrations of acid-hydrolysable (AHPI-C and AHPII-C) and recalcitrant C (RP-C) pools at low elevations indicated that high temperatures at these elevations maintained the high decomposition of SOM, resulting in less organic C accumulation. The acid-hydrolysable SOM pool has a rapid turnover rate that favors soil microbial activity [40, 43]. The large acid-hydrolysable C pools in bamboo plantations at high elevations supported a large amount of C and N in microbial biomass [22]. When considering the total organic C (TC), the AHPII-C/TC ratio was significantly decreased with increasing elevation, whereas the AHPI-C/TC ratio did not exhibit a trend with increasing elevation. The significantly higher AHPII-C/TC ratio at low elevations suggested that the release of acid-hydrolysable C from easily decomposed bamboo litter was greater at low elevations. In contrast, the RP-C/TC ratio was significantly greater at higher elevations. The result agreed with the findings of Belay-Tedla et al. [43] who showed that high soil temperature in warmed plots may stimulate decomposition of recalcitrant C pools and decreased RP-C proportion.

In addition to climatic factors, site-specific factors such as cultivation history could affect the degree of SOM humification in the bamboo plantations. The A/O-A ratio is also considered an index of the degree of humification because alkyl-C increase and O-alkyl-C decrease, relatively, with litter decomposition [38]. In general, the alkyl-C and A/O-A ratio is higher under high humification [42]. The increase in alkyl-C abundance and A/O-A ratio with increasing elevation observed in this study suggests a greater degree of SOM humification at higher rather than lower elevation. However, the aromaticity and ΔlogK results indicated a low degree of humification at high elevations. The inconsistency in estimates of SOM humification in this study might be due to the confounding effect of the remaining SOM from the original forest. Establishment of bamboo plantations in broadleaf forests took place later at high elevations (less than 60 years ago) compared with low-elevation sites (60–110 years ago) [22, 26]. Thus, the cessation of broadleaf residue inputs from the original forest occurred more recently in higher elevation bamboo plantations. The low temperatures at high elevations supported the lower rate of SOM decomposition favoring the preservation of forest broadleaf residues. In addition, long-term and intensive cultivation in low-elevation soils accelerated the decomposition of broadleaf residues. A similar result found that long-term intensive cultivation could decrease total C and labile C pools and accelerate the degradation of SOM in moso bamboo plantation [24]. Thus, it is reasonable to speculate that the A/O-A ratios in high-elevation bamboo plantations were influenced more strongly by the broadleaf residues of the original forest. Because broadleaf residues have higher A/O-A ratios (0.41) than litter from bamboo (0.18), a relatively greater amount of broadleaf residues from the original forests could account for the high A/O-A ratio in high-elevation bamboo soil.

The climatic variation in temperature and moisture along the elevation gradient greatly dominated trends in SOM pools and humification in this study. Similar patterns were reported previously [12, 44]. However, if we consider the composition of humic acids obtained from NMR spectroscopy and photometric analysis, site-specific factors such as cultivation history and the remaining SOM from original forest also play an important role in the SOM humification. Djukic et al. [6] indicated that SOM functional groups were inconsistent across an elevation gradient but were strongly affected by vegetation composition, C input and litter quality. Schindlbacher et al. [14] noted that forest management and land-use history may play an important role in soil development and decomposition under certain climatic conditions in Austria. The combined use of NMR spectroscopy and photometric analysis could provide insight into the composition of humic acids, which reflects the interactions between climatic and site-specific factors such as cultivation history and vegetation type.

## Conclusions

The positive relationships between elevation and soil total organic C and total N in moso bamboo plantations showed that the altitudinal changes in climatic conditions (i.e., temperature) could have a critical effect on the rate of decomposition and SOM accumulation. Both the aromaticity and ΔlogK indicated that the degree of humification decreased with increasing elevation. However, the alkyl-C and A/O-A ratio increased with increasing elevation, possibly because of a larger amount of SOM from the original broadleaf residues at high elevations. The positive relation between the RP-C/TC ratio and elevation also indicated that high temperatures and intensive bamboo management at low elevations could have accelerated the decomposition of recalcitrant fractions. This study suggests that altitude controls the degree of SOM humification in these bamboo plantations; however, humic acid composition and SOM humification were also controlled by site-specific factors such as antecedent SOM and cultivation history.

## Supporting Information

S1 Fig Land use in 1904. (JPG)

S2 Fig Land use in 1956. (JPG)

S3 Fig Land use in 2006. (JPG)
