# Supplementary figures and images for: Humic Acid Composition and Characteristics of Soil Organic Matter in Relation to the Elevation Gradient of Moso Bamboo Plantations
Source: PLoS One. 2016 Sep 1;11(9):e0162193. doi: 10.1371/journal.pone.0162193 (PMC5008779; doi:10.1371/journal.pone.0162193)

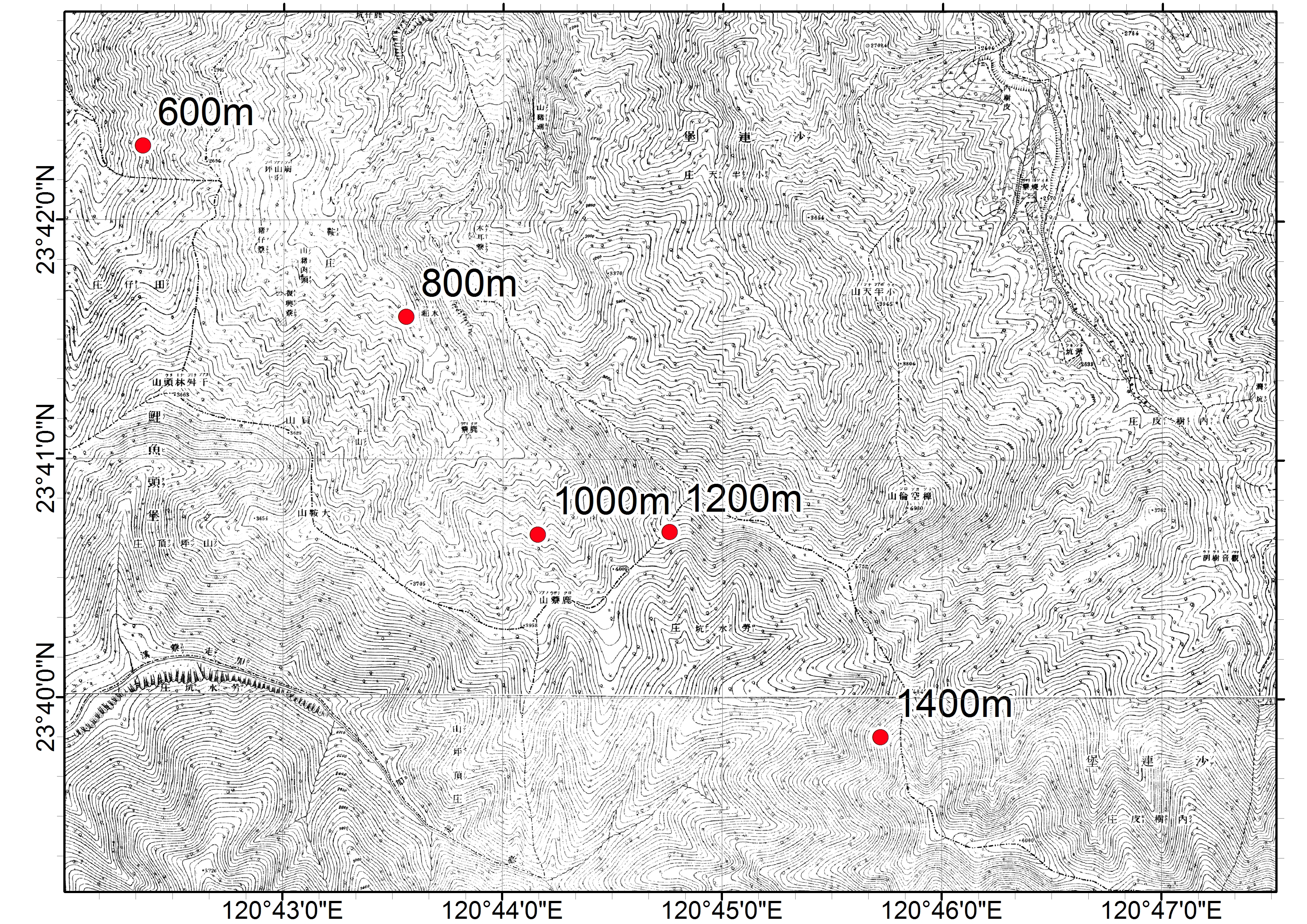

Supplement: S1 Fig — (JPG) [file pone.0162193.s001.jpg]

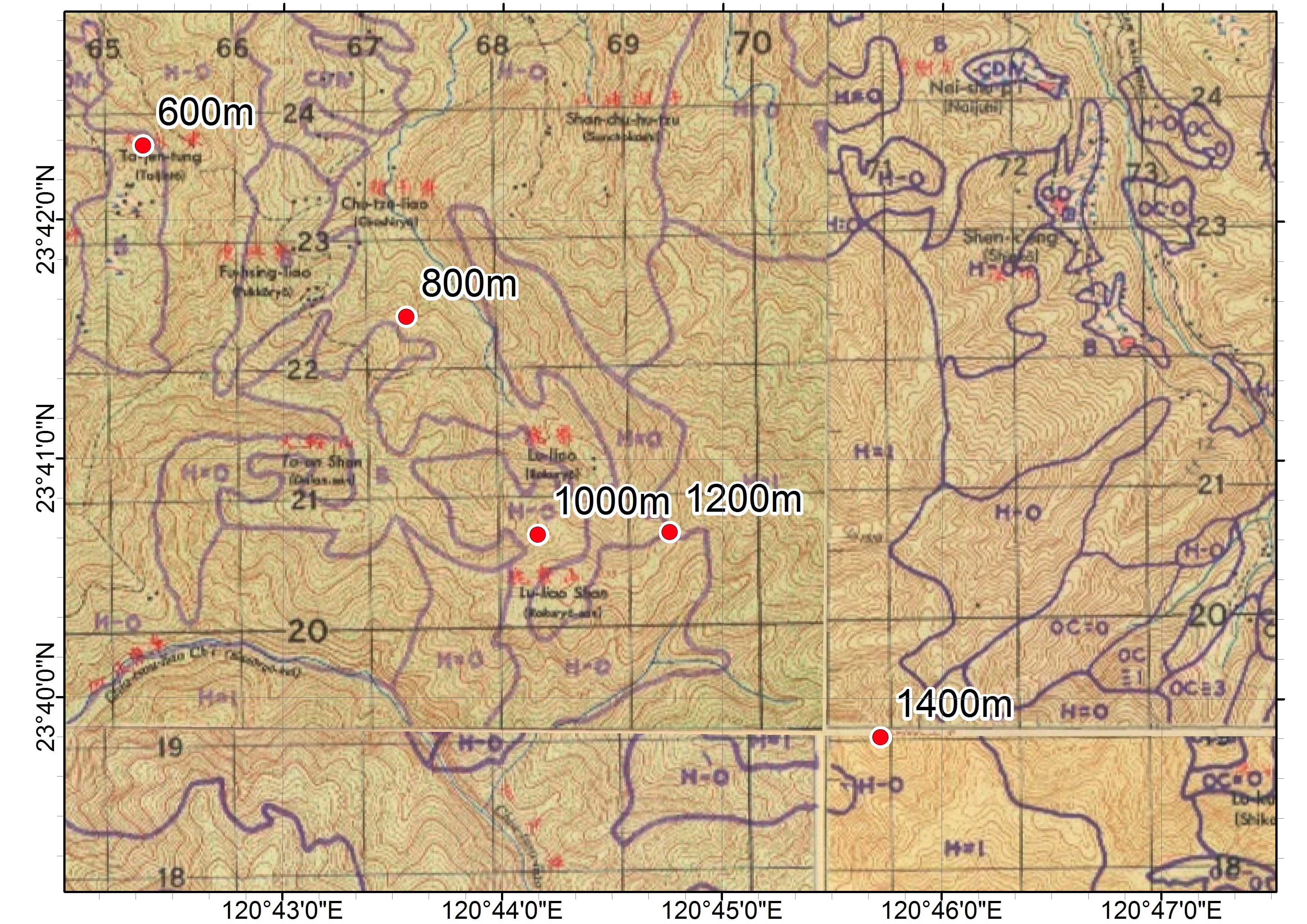

Supplement: S2 Fig — (JPG) [file pone.0162193.s002.jpg]

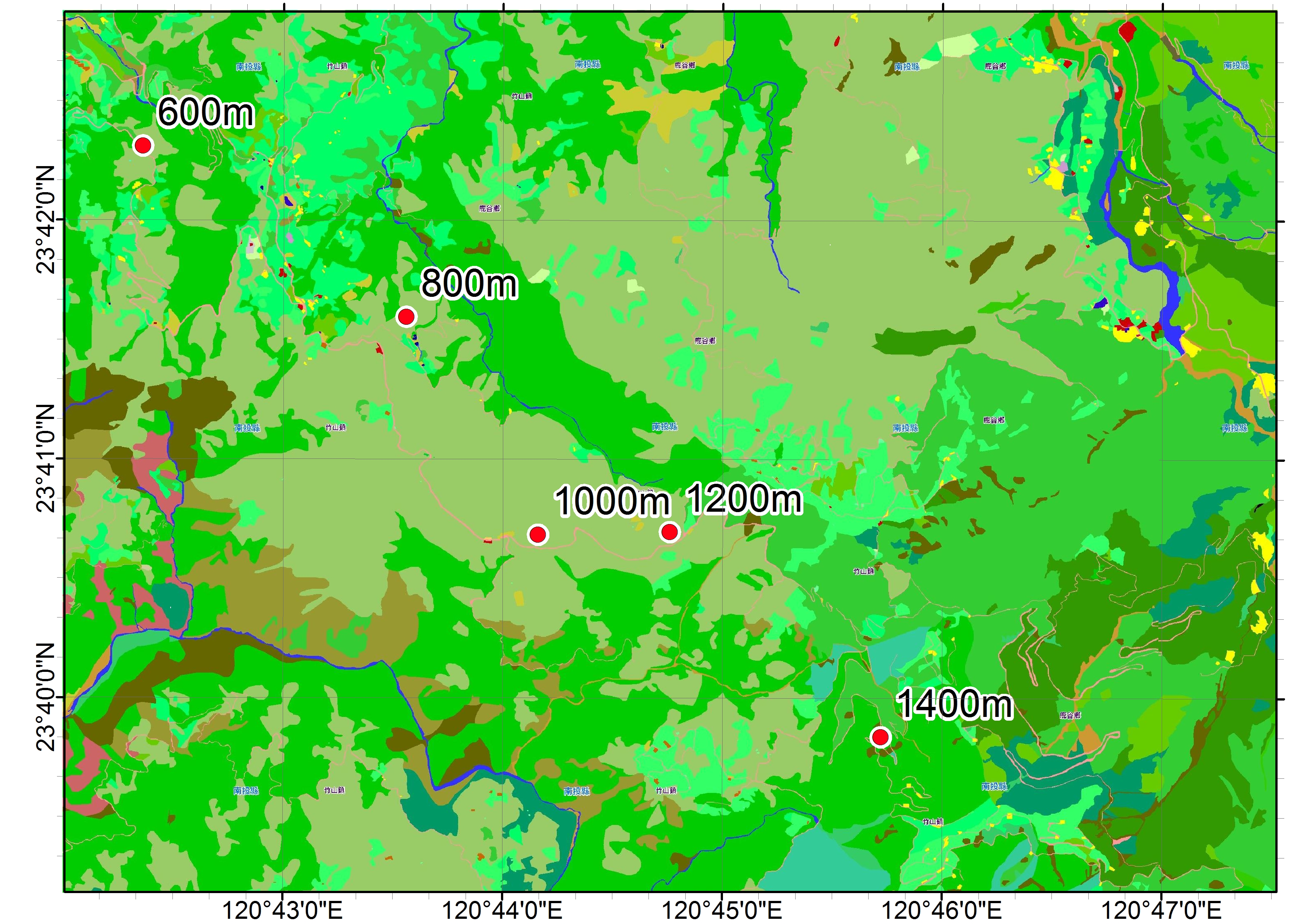

Supplement: S3 Fig — (JPG) [file pone.0162193.s003.jpg]
